# Homozygous Recessive Versican Missense Variation Is Associated With Early Teeth Loss in a Pakistani Family

**DOI:** 10.3389/fgene.2018.00723

**Published:** 2019-01-21

**Authors:** Stefania Bigoni, Marcella Neri, Chiara Scotton, Roberto Farina, Patrizia Sabatelli, Chongyi Jiang, Jianguo Zhang, Maria Sofia Falzarano, Rachele Rossi, Davide Ognibene, Rita Selvatici, Francesca Gualandi, Dieter Bosshardt, Paolo Perri, Claudio Campa, Francesco Brancati, Marco Salvatore, Maria Chiara De Stefano, Domenica Taruscio, Leonardo Trombelli, Mingyan Fang, Alessandra Ferlini

**Affiliations:** ^1^Medical Genetics Unit, Department of Medical Sciences, University of Ferrara, Ferrara, Italy; ^2^Research Centre for the Study of Periodontal and Peri-Implant Diseases, University of Ferrara, Ferrara, Italy; ^3^Institute of Molecular Genetics, National Research Council of Italy, Bologna, Italy; ^4^BGI-Shenzhen, Shenzhen, China; ^5^Department of Periodontology and Department of Oral Surgery and Stomatology, School of Dental Medicine, University of Bern, Bern, Switzerland; ^6^Eye Clinic, Sant’Anna University Hospital, Ferrara, Italy; ^7^Department of Life, Health and Environmental Sciences, University of L’Aquila, L’Aquila Italy; ^8^Laboratory of Molecular and Cell Biology, Istituto Dermopatico dell’Immacolata, IDI-IRCCS, Rome, Italy; ^9^National Institute of Health, Rome, Italy; ^10^Division of Clinical Immunology, Department of Laboratory Medicine, Karolinska Institute at Karolinska University Hospital Huddinge, Stockholm, Sweden; ^11^Dubowitz Neuromuscular Unit, University College London, London, United Kingdom

**Keywords:** versican, human, periodontium, dental cementum, Wagner syndrome

## Abstract

Only a few genes involved in teeth development and morphology are known to be responsible for tooth abnormalities in Mendelian-inherited diseases. We studied an inbred family of Pakistani origin in which two first-cousin born brothers are affected by early tooth loss with peculiar teeth abnormalities characterized by the absence of cementum formation. Whole exome sequencing revealed a H2665L homozygous sequence variant in the *VCAN* gene. Dominant splicing mutations in *VCAN* are known to cause Wagner syndrome or vitreoretinopathy. We explored teeth morphology in these two patients, while versican expression was assessed by western blot analysis. Early signs of vitreoretinopathy were found in the elder brother while the parents were completely negative. Our findings suggest that the homozygous recessive H2665L missense sequence variant impairs the normal morphology of the teeth roots via loss of cementum synthesis, and is also associated with early onset, recessive, Wagner syndrome, thus expanding both the phenotype mutation scenario and the inheritance mode of *VCAN* mutations.

## Introduction

Non-syndromic dental diseases can be classified as diseases affecting the dental formula (e.g., tooth agenesis, hyperdontia) and diseases affecting the shape and structural characteristics of the tooth (Bailleul-Forestier et al., 2008; Galluccio et al., 2012; Klein et al., 2013). The genes associated with early tooth loss are not yet completely known. While these features may be present in some known genetic syndromes (for example Down Syndrome, Chediak-Higashi Syndrome, Papillon-Lefevre Syndrome), only odontohypophosphatasia, a genetic autosomal recessive condition due to specific mutations in the *ALPL* gene (OMIM 146300), represents, to our knowledge, the only disease known to be characterized by isolated premature exfoliation of primary and/or permanent teeth (often associated with severe dental caries) in the absence of any skeletal or systemic abnormalities.

No other genes, to date, are associated with this peculiar developmental teeth anomaly. In the present study, we report the first case of a novel, homozygous, recessive, missense sequence variant in the *VCAN* gene (Chondroitin sulfate proteoglycan 2, versican or *CSPG2* gene NM_004385.4), identified by whole exome sequencing (WES), in two young brothers with a developmental anomaly of the dental and periodontal structures which was associated with spontaneous tooth exfoliation. The developmental anomaly consisted of the partial to complete lack of the root complex, the presence of dysplastic dentine and the complete absence of cementum and periodontal ligament tissue. *VCAN* mutations are known to cause Wagner syndrome (OMIM #143200), an autosomal dominant disease characterized by isolated vitreoretinopathy. Interestingly, the 8 reported mutations are invariably occurring at splice sites or consensus sequences affecting splicing. These known mutations occur in intron 7 (c.4004-2A > T; c.4004-2A > G; c.4004-5T > C; c.4004-5T > A; c.4004-1G > A; and c.4004-1G > C) and in intron 8 (c.9265+1G > A; and c.9265+2T > A). Recently, a deletion involving *VCAN* gene exon 8 was also described in a Wagner family (Burin-des-Roziers et al., 2017).

In our family, the sequence variant is the missense H2665L and it is in homozygosis. The sequence variant lies in the chondroitin sulfate attachment region β domain and is expected to affect the V0 and V1 VCAN isoforms.

It is worth noting that the dental phenotype in our family is also associated with an initial vitreoretinopathy in the elder brother. We conclude that *VCAN* mutations may cause very different phenotypes which could possibly overlap if mutations are recessive and affect the V0 full-length isoform or, alternatively, show a different tissue targeting and specific clinical features, if dominant. This is also an additional example of a disease-causing gene exhibiting pathological consequences by both recessive and dominant fashions.

## Materials and Methods

### Ethics Statement

The study was approved by the Ethical Committee of the University of Ferrara, Italy (protocol n. 139-2012, date of approval: December 20, 2012). Written informed consent was obtained from the parents of the examined subjects both for the study participation and for the publication of this case report.

### Medical and Dental History

**FIGURE 1:**
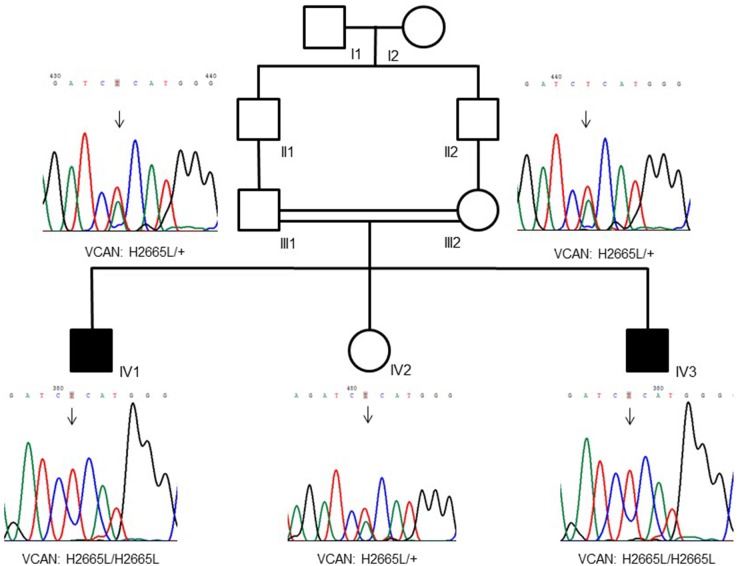
Family pedigree showing a recessive inheritance model of the VCAN sequence variant (p.H2665L). Consanguinity is also visible in the tree.

The family (pedigree in Figure 1) originates from Pakistan and immigrated to Italy. Parents were first cousins and healthy, and referred their two sons (7 and 2 years old) for a dysmorphology evaluation due to the loss of several teeth by spontaneous exfoliation just a few months after their eruption. No past or current systemic diseases emerged from medical anamnesis that could be associated with early spontaneous tooth exfoliation.

Skeletal x-ray surveys, performed in the elder brother, did not reveal bony alterations suggestive of any syndromic condition involving the skeleton characterized by the premature loss of teeth.

Biochemical parameters such as serum calcium and phosphorus, alkaline phosphatase, LDH, serum and urinary phosphatidylethanolamine were evaluated in the elder brother in order to exclude odontohypophosphatasia, which were in the normal range. *ALPL* gene sequencing did not reveal any pathological variation.

### Oral Inspection and Orthopantomographic Exams of the Index Case (7-Year-Old Son)

At the first dental visit (June 2012), the 7-year-old son was examined extra-orally and intra-orally. In particular, a comprehensive examination of the mucosal, dental and periodontal structures was performed. During the extraction of some teeth that were symptomatic due to pronounced (grade III) mobility, gingival tissue samples were collected from adjacent areas and stored for laboratory analyses.

Orthopantomographic exams performed in 2009 and 2012 were also compared for dental formula and alveolar bone levels.

### Ophthalmological Evaluation

Members of the quartet (brothers and parents) underwent a standard ophthalmological evaluation. In the brothers’ common stereo tests (Lang I and II, TNO, TITMUS, and FRISBY), intraocular pressure detection, biomicroscopy, indirect ophthalmoscopy and ocular ultrasonography were also performed.

Optical coherence tomography (OCT, Spectralis, Heidelberg Engineering, Germany) and visual field tests (30-2, ZEISS Humphrey Field Analyzer 3, Germany) were performed only in the elder brother.

### Histological Analysis

Five teeth were lost in the period between June 2012 and October 2013 and were stored in 10% buffered formalin immediately after their exfoliation and sent to the Robert K. Schenk Laboratory of Oral Histology, University of Bern, for histological analysis. Two teeth were decalcified in 10% EDTA, subdivided into smaller pieces and embedded in LR White resin and paraffin. Three teeth were processed for the production of undecalcified ground sections. The specimens were briefly rinsed under running tap water, dehydrated in ascending concentrations of ethanol, and embedded in methyl methacrylate. The embedded tissue blocks were cut parallel to the central axis of the teeth into 400 μm-thick ground sections using a slow-speed diamond saw (Varicut^®^VC-50, Leco, Munich, Germany). After mounting the sections onto acrylic glass slabs, they were ground and polished to a final thickness of about 100 μm (Knuth-Rotor-3, Struers, Rodovre/Copenhagen, Denmark) and the surfaces stained with toluidine blue/McNeal and basic fuchsin (Schenk et al., 1984). Digital photography was performed using a Zeiss AxioCam MRc camera (Carl Zeiss, Göttingen, Germany) connected to a Zeiss Axio Imager M2 microscope (Carl Zeiss).

### Whole Exome Sequencing and Data Analysis

Genomic DNA was extracted from peripheral blood with standard protocols and WES was performed on both the affected brothers and their normal parents at BGI-Shenzhen using the NimbleGen SeqCap EZ Human Exome Library v3.0 (Nimblegen 64 Mb) exome capture kit (NimbleGen, Madison, WI, United States) according to the manufacturer’s instructions as previously described (Schindler et al., 2016), and enriched libraries were sequenced by Illumina Hiseq2000 platform (Illumina, Inc. San Diego, CA, United States) with 90-bp paired-end reads. Sequencing reads were aligned to the human reference genome (NCBI build 37.1, hg19) using both SOAPaligner and BWA with default parameter. SNVs and indels were detected by using SOAPsnp (soap2.21) (Li et al., 2009) and GATK (version v1.0.4705) (McKenna et al., 2010). Polymorphic sites of detected variants were filtered by three public databases, including the 1000 Genomes Project^1^, ESP^2^, ExAC^3^ with MAF > 0.5% and BGI-In-house databases. Since the parents were consanguineous, we used homozygosity mapping analysis (Lamperti et al., 2012) to first detect the homozygous region narrowing down the candidate gene list.

### Biopsies and Cell Cultures

A gingival biopsy was obtained from the two affected brothers (individuals IV-1 and IV-3 in Figure 1) and from two age- and sex-matched control subjects. Biopsies were subjected to mechanical dissociation for gingival fibroblast cultures. Cells were maintained in Dulbecco’s Modified Eagle Medium (DMEM) containing 1% antibiotics plus 10% Fetal Bovine Serum (FBS).

### Western Blot

Proliferating and confluent cultured gingival fibroblasts were harvested by scraping, and cell lysates were resolved by standard SDS–PAGE then electro-blotted onto a nitrocellulose membrane and incubated with antibodies against *VCAN* (Novus Biologicals) and actin (Sigma–Aldrich) as a loading control, followed by incubation with anti-mouse or anti-rabbit horseradish peroxidase (HRP)-conjugated secondary antibodies. Chemiluminescent detection of proteins was carried out with the ECL Detection Reagent Kit (GE Healthcare Amersham, Pittsburgh, PA, United States) according to the supplier’s instructions.

## Results

### Oral Inspection and Orthopantomographic Exams of the Index Case (7-Year-Old Son)

**FIGURE 2:**
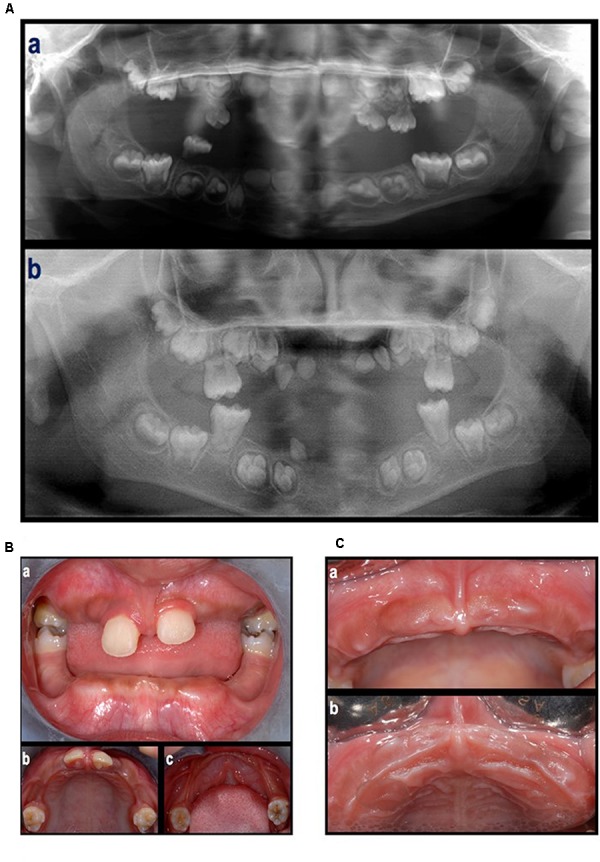
**(A)** An orthopantomograph taken in 2009 **(a)**, tooth germs of 1.7, 1.6, 1.5, 1.4, 1.3, 1.2, 1.1, 2.1, 2.2, 2.3, 2.4, 2.5, 2.6, 2.7, 3.7, 3.6, 3.5, 3.4, a tooth germ in region 4.3, two tooth germs in region 4.1–4.2, and tooth germs of 4.3, 4.4, 4.5, 4.6, and 4.7, were present. The germs of the first molars showed incomplete root development. The presence of deciduous teeth was observed in the right maxillary quadrant (1 molar), left maxillary quadrant (two molars and one canine), and right mandibular quadrant (1 molar). All teeth showed signs of extensive root resorption. An orthopantomograph taken in 2012 **(b)**, the four first molars were erupted and showed partial (maxilla) to complete (mandibula) root development. Tooth germs of 1.8, 1.7, 1.5, 1.4, 2.4, 2.5, 2.7, 2.8, 3.8, 3.7, 3.5, 3.4, 4.4, 4.5, 4.7, and 4.8, were present. Four teeth in the anterior region of the maxilla (probably 1.3, 1.2, 2.2, and 2.3) and one tooth in the right canine region of the mandible (probably 4.3), all characterized by limited crown dimension as well as limited to absent root development, were present. None of them were fully erupted. **(B)** Intra-oral, frontal **(a)** and occlusal **(b,c)** view at first visit (June, 2012). **(C)** Clinical aspect of the maxillary arch after the loss of the maxillary central incisors (September, 2012).

In the first visit (June 2012), extra-oral examination did not reveal evident asymmetries or disproportions among the upper, middle, and lower thirds of the face. In addition, lateral views showed a harmonic face profile. Intra-oral examination showed no lesions or pathologic conditions affecting oral mucosae. Four permanent teeth (two molars and two central incisors) were present in the maxillary arch and two permanent molars in the mandibular arch (Figure 2A). All teeth showed a mobility ranging from degree 1 to 3 (Carranza and Newman, 1996). A slight, plaque-induced gingival inflammation (redness of the gingival margin, bleeding on probing) was present at the buccal aspect of tooth 1.1. No sites with clinical attachment loss were present and probing depths were ≤ 4 mm. No carious or endodontic lesions were diagnosed. The occlusal view of the dental arches showed a cutting-edge profile of the crest in the edentulous regions. In 1 year time, the patient lost five permanent teeth (2 maxillary central incisors, 2 maxillary first molars, and 1 canine) by spontaneous exfoliation. The root complex of exfoliated teeth was almost completely absent or limited to the coronal third.

Panoramic radiographs showed that all deciduous teeth and five permanent teeth (2 maxillary central incisors and 3 teeth in the mandibular incisor/canine region) were lost (Figures 2B,C).

### Histological Analysis

**FIGURE 3:**
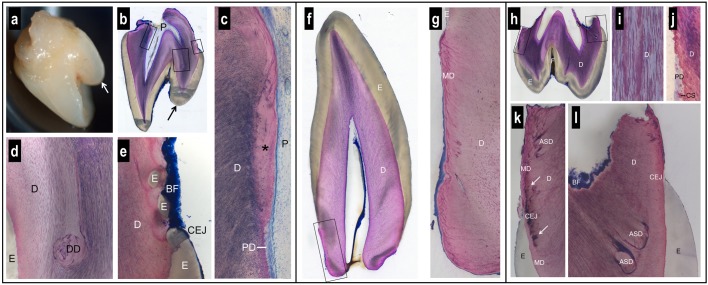
Macroscopic and histologic views of spontaneously exfoliated teeth. Maxillary central incisor **(a–e)**. Note the prominent supplementary palatal cusp (arrow) and the very short, blunt root **(a,b)**. Biofilm (BF) is present on the crown and root surface, but root cementum is missing **(b,e)**. Coronal to the level of the cementum-enamel junction (CEJ), the dental pulp (P) and dentin (D) look normal **(b)**. Apical to the CEJ, there is a lack of dentinal tubules in the most recently formed dentin (^∗^) and a predentin (PD) layer is missing **(c)**. In one region, dysplastic dentin (DD) was observed with a reduced number of dentinal tubules **(d)**. Apical to the palatal CEJ, small islands of enamel (E) were seen surrounded by dentin **(e)**. Canine **(f,g)**. The anatomy of the crown and the morphology of enamel (E) and dentin (D) appear normal **(f)**. However, the root is short, blunt, very conical, and free of signs of resorption **(f)**. The peripheral dentin exhibits a low number of dentinal tubules, an abnormal structure of mantle dentin (MD), and no cementum **(g)**. Maxillary first molar **(h–l)**. The tooth has a normal enamel (E) layer with a deep fissure (F) and short, partially resorbed roots **(h)**. The dentin (D) exhibits an irregular numerical density of dentinal tubules **(i)**. Apical to the level of the cementum-enamel junction (CEJ), a predentin (PD) layer and typical calcospherites (CS) are missing **(j)**. Altered dentin morphology is seen close to the CEJ with darkly stained matrix (arrows) in the region of the mantle dentin (MD) **(k)**, and dysplastic, arcade-shaped dentin (ASD) with a reduced number of dentinal tubules **(l)**. Biofilm (BF) covers the surface of the resorbed root trunk. A cementum layer is absent **(k,l)**.

The first maxillary right incisor presented, in addition to its normal incisal edge, a prominent supplementary palatal cusp, known as a talon cusp (Figures 3a,b). The crown anatomies of the canine (either mandibular or maxillary) (Figure 3f) and maxillary first molar (Figure 3h) were inconspicuous, with the exception of a rather deep enamel fissure in the molar.

While enamel thickness and morphology were normal in all teeth, striking alterations were observed with regard to the anatomy of the roots and the morphology of the dentin. The roots were short, blunt and concave, i.e., inward bending (Figures 3a,b,f,h). While clear signs of root resorption were evident in one tooth (Figure 3h), the two other teeth did not reveal resorption cavities (Figures 3b,f). Biofilm was deposited on the root dentin of two teeth (Figures 3b,e,h,k,l). In all teeth, there was no cementum layer detected on the root dentin or elsewhere.

Dysplastic dentin in the form of arcade-shaped structures was detected in the region of the mantle dentin at the cemento-enamel junction level. The number of dentinal tubules was lower in this dysplastic dentin than in the surrounding dentin (Figure 3l). Following the course of the dentinal tubules, from the dysplastic dentin to the dental pulp, revealed that a predentin layer and the calcospherites at the mineralization front were missing from this site apically (Figure 3j). In one tooth, the dentin at this site was devoid of dentinal tubules (Figure 3c). An irregular number of dentinal tubules was observed in many regions of the dentin (Figures 3d,g,i). In one tooth, islands of enamel were found surrounded by dentin, apical to the cemento-enamel junction (Figure 3e).

### Whole Exome Sequencing and Data Analysis

WES was performed on both the affected siblings and their normal parents (family-of-four strategy). Approximately 121766 SNVs and 23892 indels were detected per sample. Seventeen shared genes with homozygous variants in both affected brothers and heterozygous states in both normal parents were interrogated after only rare amino acid changing variants were kept. The number of genes was reduced to 14 after considering the homozygous region for both patients (Supplementary Table S1). We prioritized the variations based on the commonly shared genomic regions (inbreeding) and allele frequency (less than 0.5%). After the filtering process, we identified the *VCAN* gene as the best candidate with a homozygous missense sequence variant in exon 8 (c.7994A > T; p.His2665Leu; chromosome 5: 82836816; GRCh37/hg19) (NM_004385.4). The homozygous sequence variant is present in both affected brothers and is heterozygous in parents and in the healthy sister. The variation MAF is = 0 in ExAc and 1000G databases, and it is only reported once in gnomAD^4^ in a heterozygous genotype not associated with a diseased status.

**FIGURE 4:**
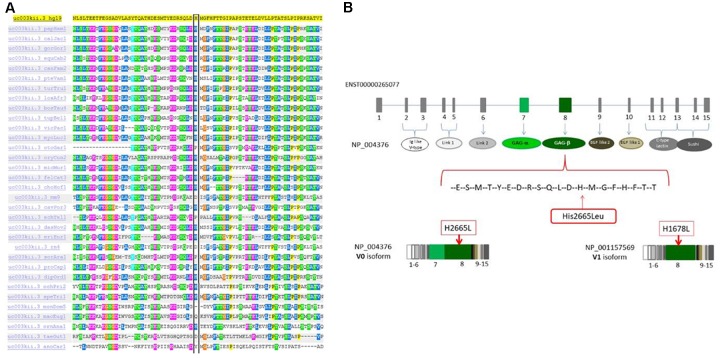
**(A)** The p.H2665L sequence variant occurred in an amino acid residue (His) that is highly conserved through different species. **(B)** Mutation localization in the protein domain scheme. The full-length protein (NP004376, isoform V0) contains 7 different domains and 2 functional regions. The domains are: Ig like V-type (ex2-ex3), Link1 (ex4-ex5), Link2 (ex6), EGF like1 (ex9), EGF like2 calcium binding (ex10), C-type lectin (ex11-ex13), sushi (ex13-ex15). The 2 glucosaminoglycan attachment domains are located in exon 7 GAα and in exon 8 GAβ. Isoform V1 (NP001157569) is similar in structure to isoform V0 but it doesn’t have the GAG-α region.

This missense variation is not concordantly predicted as pathogenic by *in silico* tools (see in Supplementary Table S2). Nevertheless, many tools are concordant in predicting H2665L as deleterious. One important clue supporting its pathogenic role in our patients is that this variant is located in the highly homozygous region inherited from both patients according to the homozygous mapping result. Moreover, the His2665 is a highly conserved amino acid through species and located in the C-terminus of the protein (Figure 4A). This amino acid change is located in exon 8, encoding the glycosaminoglycan attachment domain GAG β (or chondroitin sulfate attachment region β). It is therefore predicted to affect the protein anchorage to the glycosaminoglycans and involves the VCAN isoforms V0 and V1 only (Figure 4B and Supplementary Figure S1).

All sequencing data information obtained by the family WES studies have been uploaded as FastQ files and are accessible in https://db.cngb.org/cnsa/search/?q=CNP0000163.

### Western Blot Analysis

**FIGURE 5:**
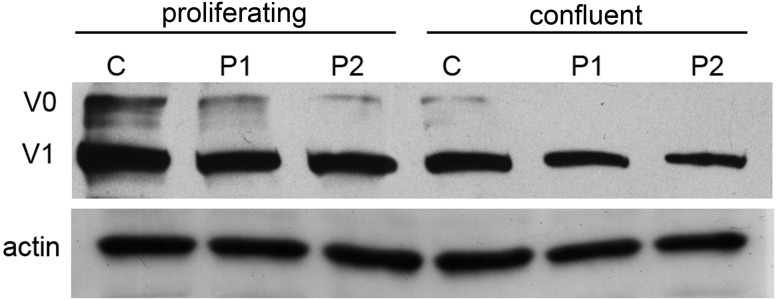
Western blot analysis of proliferating and confluent cell cultures from normal (C) and patient 1 and 2 (P1 and P2) of VCAN (V0 and V1 isoforms). Actin was used as a loading control. Normalization of densitometric values of V0 and V1 on actin in proliferating (V0, 1.28, 0.84, 0.82; V1, 0.86, 0.7, 0.59 in control, P1 and P2, respectively) and confluent cells (V0, 0.52, 0.27, 0.17; V1, 0.19, 0.2, 0.23 in control, P1 and P2, respectively) shows a clear reduction of VCAN V0 and V1 isoforms in patient cell lysates under proliferating conditions. Due to secretions induced by cell–cell contact, in confluent samples both V0 and V1 isoforms are quantitatively reduced with respect to the proliferating conditions, both in control and patient samples.

Western blot analysis revealed a clear reduction of the V0 and V1 VCAN isoforms in proliferating cell lysates of both patients, with the V0 isoform appearing quantitatively more affected when compared with control cells. These two isoforms are expected to be affected by the H2665L variation. In confluent cultures, a moderate reduction of the V1 isoform was confirmed in patient cultures, while the V0 isoform was faintly detected in both control and patient cells, due to the effect of cell density on *VCAN* regulation and full-length protein secretion into the culture medium (Zimmermann et al., 1994). On the contrary, actin, used as a loading control, revealed a comparable amount of protein in patient cultures with respect to normal cells (Figure 5).

### Ophthalmological Evaluation

**FIGURE 6:**
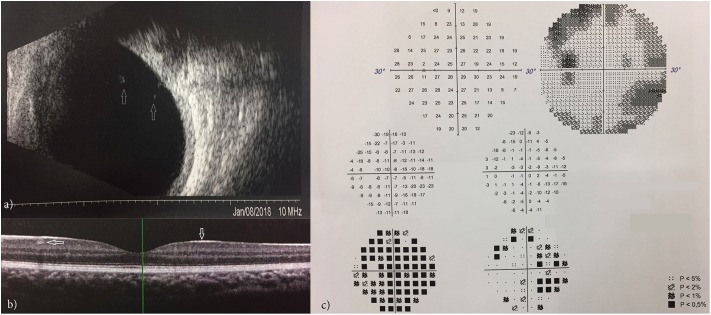
Ocular ultrasonography showing vitreous condensation (white arrows) **(a)**; OCT showing a hyperreflective inner limiting membrane with hyperreflective foci within the ganglion cell layer **(b)**; VFT showing points of reduced retinal sensitivity **(c)**.

Eye examination of the elder brother disclosed a reduced visual acuity in both eyes (20/25 in the right and 20/30 in the left) in the absence of any refractive error. This patient presented with only a mild exophoria at near and far distances but a complete lack of stereopsis: in fact he could not perform any of the common stereo tests (Lang I and II, TNO, TITMUS and FRISBY). Ocular anterior segment and intraocular pressure were normal; indirect ophthalmoscopy revealed bilaterally minimal peripheral vitreous condensation which was also documented by ocular ultrasonography (Figure 6a). OCT (Spectralis, Heidelberg Engineering, Germany) of the macula in both eyes disclosed a particularly hyperreflective inner limiting membrane with hyperreflective foci within the ganglion cell layer (Figure 6b). Visual field tests (30-2, ZEISS Humphrey Field Analyzer 3, Germany) showed several points of reduced retinal sensitivity bilaterally (Figure 6c). Ophthalmologic examination of the younger brother was normal, with a minimal amount of peripheral vitreous condensation. Typical signs of vitreoretinopathy were absent in both parents, excluding the presence of a vitreous or retinal disease, even at a subclinical stage.

## Discussion

Anomalies of tooth structure or development can frequently be part of syndromic defects and rarely occur as unique signs of diseases. Indeed, only a few disease genes are known causing tooth agenesis, anodontia, or developmental anomalies of the dental structures (Bei, 2009; Frazier-Bowers and Vora, 2017).

Here we report a consanguineous Pakistani family with two young brothers affected by early tooth loss. The main dental anomaly is morphological and characterized by the complete absence of cementum as shown in the histological analysis of the teeth, causing premature tooth loss. A complete lack of dentinal tubules in the dentin and predentin is also evident, together with dysplastic, arcade-shaped dentin with all defects supporting a developmental defect. The two brothers do not show any other major signs or symptoms, clinical examination was normal, excluding the presence of a syndromic condition.

We performed WES on this quartet and identified a homozygous recessive missense variation in the *VCAN* gene (c.7994A > T, NP_004376: p.H2665L). This variation was never reported in homozygosis and is present only once in gnomAD in a heterozygous genotype. It has never been associated with any disease, therefore it can be defined as a very rare variant. It occurs in a highly conserved domain of the VCAN protein. *In silico* analysis predicts that it could possibly be pathogenic, but it is also well known that *in silico* predictors should be used with caution since each of them has a certain percentage of false positive and false negatives. Notably, H2665L variant resides in a cistronic (non-recombinant) unit located in the homozygous region of both patients, according to the WES homozygous mapping result. This finding strongly supports the involvement of the VCAN locus containing the H2665L variation in our family, with a recessive inheritance model.

Versican (*VCAN*, OMIM ^∗^118661) consists of 15 exons and encodes 4 different isoforms (V0 to V3) sharing the NH2 and COOH terminus but varies in the chondroitin sulfate binding domains (alpha and/or beta), which are missing in isoform V3 (Supplementary Figure S1). The full-length mRNA is 12361 bp long. The mammalian VCAN protein is a large chondroitin sulfate proteoglycan expressed during chondrogenesis and a major component of the extracellular matrix. The protein has a highly versatile function, as the eponym refers (Bei, 2009). It is involved in cell adhesion, proliferation, migration and angiogenesis and plays a central role in tissue morphogenesis and maintenance.

In mouse dental tissue, VCAN expression occurs primarily in mesenchymal tissue as well as in epithelial tissue. The expression of VCAN mRNA is not the same in the different phases of teeth development. It has been shown to appear first in the thickened dental epithelium (12 weeks) and then it continues to be expressed in the enamel organ until the bell stage (Jiang et al., 2010). Versican was detected by immunostaining in the stellate reticulum areas from the bud stage to the apposition stage. The enamel organ (16 weeks) expressed VCAN mRNA at a level comparable to that in dental mesenchyme.

In fully developed human teeth, VCAN fragments are significant constituents of the human dentine and predentine organic matrix, while VCAN whole molecules can be visualized in scarce amounts within predentine only (Orsini et al., 2007). Finally, intensive immunostaining for VCAN was found almost exclusively in the lacunae housing cementocytes in cementum and osteocytes in alveolar bone, respectively, suggesting an important, though still unrevealed, role of VCAN in cementogenesis (Ruggeri et al., 2009). *VCAN* mutations have never been reported as being associated with teeth anomalies in humans, accordingly to literature and OMIM (^∗^118661). Nevertheless, mutations in the *VCAN* gene are known to cause Wagner syndrome (OMIM #143200), a very rare dominant disease-causing vitreoretinopathy. Within the eye, *VCAN* interacts with other proteins to maintain the structure and gel-like consistency of the vitreous (Theocharis et al., 2002).

Wagner syndrome is mainly due to very rare mutations affecting VCAN isoforms splicing with a dominant inheritance. A few deletions were also reported (Burin-des-Roziers et al., 2017). Teeth abnormalities were never described in Wagner syndrome.

The homozygous missense variation we have identified in our family is located in the GAG-beta domain affecting isoforms V0 (full length) and V1. Cementum is completely absent in our patients’ teeth, as visible from the tooth morphological analysis; peripheral dentin exhibits a low number of dentinal tubules and an abnormal structure of mantle dentin. All these signs strongly suggest a developmental anomaly (Hou et al., 2012). Strikingly, western blotting showed a clear reduction of VCAN V0 and V1 isoforms in patient gingival fibroblasts, especially in proliferating cells, a culture condition which promotes VCAN protein synthesis (Zimmermann et al., 1994). V0 is the full-length isoform containing the GAG-beta domain where the H2665L is located. Although *VCAN* mutations were never described in humans causing tooth disorders, VCAN is known to have a role in dentinogenesis and cementogenesis in animals. In mice, *VCAN* is indeed expressed in dental pulp, odontoblasts, cementoblasts, cementocytes, periodontal ligament cells, osteoblasts and osteocytes, and its spatio-temporal expression is regulated by ADAMT proteins (Sone et al., 2005). By RNA *in situ* hybridization, *VCAN* was observed as developmentally regulated during ontogenesis, being expressed first in enamel until the bell stage together with a quartet of proteins, Dspp, Mepe, and Mimecan, which complementary orchestrate the odontogenesis process (Jiang et al., 2010).

More than 300 genes are known to orchestrate human tooth formation (Bei, 2009). These genes belong to five main signaling pathways: bone morphogenetic proteins, fibroblast growth factor, Notch signaling, sonic hedgehog proteins and wingless/integration 1 signals. During tooth organogenesis, the interplay between epithelium and mesenchyme regulates cell differentiation and, consequently, the formation of distinct anatomical and functional parts of the tooth. Cementum is believed to play a regulatory role in periodontal regeneration through a variety of extracellular matrix macromolecules, as the proteoglycans. *VCAN* is expressed in cementocytes but absent in acellular cementum. We have seen that its reduction (quanti-qualitative) in patient gingiva may alter the activity of the specific cementocyte populations deputed to maintain the periodontal regeneration at the cementum-enamel junction via a fine dialoguing with other extracellular matrix proteins. Indeed, the patients show irregular numerical density of dentinal tubules, abnormal structure of mantle dentin and all structures located at this junction.

Interestingly, *VCAN* is under the control of the Wnt/beta cathenin signaling pathway (Martínez-Moreno et al., 2012). Activation of Wnt/beta cathenin via nuclear translocation of beta-cathenin induced by calcitriol, a vitamin D receptor agonist, induces upregulation of *VCAN*. This finding highlights that *VCAN* expression is also orchestrated by several external factors, and also opens speculative reflections about possible treatment using Vitamin D in our patients.

Further phenotype examination in the two brothers showed that early signs of vitreoretinopathy are present in the elder boy, suggesting that this missense variant may be causing Wagner syndrome. Interestingly, the two heterozygous parents resulted negative at the ocular examination, implying that in this family, the vitreoretinopathy is also due to a homozygous genotype. This suggests that the Wagner syndrome-related phenotype in this family is recessively inherited and possibly occurs with an earlier onset and more severe phenotypes, being already present in the 9-year-old boy. These ocular signs should certainly be monitored further via patients and parents follow-up, but the ocular signs we observed in this boy, such as hyperreflective inner limiting membrane and foci within the ganglion cell layer and reduced retinal sensitivity, are typical of the vitreoretinopathy occurring in Wagner syndrome (Thomas et al., 2016).

## Conclusion

We identified a novel homozygous recessive missense variation in the *VCAN* gene associated with tooth abnormalities in two brothers of a consanguineous family. The siblings show complete absence of cementum; the elder brother (now age 9) also shows early signs of vitreoretinopathy. The H2665L sequence variant is predicted to affect the full-length V0 isoform and therefore possibly altering all VCAN functions in both the teeth and eyes. We speculate that VCAN is a key gene involved in teeth development via cementocytes that serve to keep periodontal regeneration active and finely balanced. We also hypothesize that Vitamin D might be considered an adjuvant drug in this disease.

We suggest that the H2665L VCAN variant causes a new recessive phenotype, opening new directions in the understanding of teeth development. *VCAN* gene also represents a further example of a disease gene, causing both dominant and recessive phenotypes.

## Author Contributions

SB, MN, RF, PP, CC, and LT performed all the clinical examinations in the family quartet. CS, RR, RS, CJ, and JZ performed the genetic analysis procedures. MSF and PS performed the cell and tissue studies. DB performed the histological studies. FG, MF, and AF revised the manuscript. FB, MS, MDS, and DT are member of the Undiagnosed Disease Network and supported the study. DO edited the manuscript. MF, LT, DB, PS, and AF interpreted the data. AF conceived the work and wrote the manuscript.

## Conflict of Interest Statement

The authors declare that the research was conducted in the absence of any commercial or financial relationships that could be construed as a potential conflict of interest.

## Footnotes

1 http://www.1000genomes.org/

2 https://esp.gs.washington.edu/drupal/

3 http://exac.broadinstitute.org/

4 http://gnomad.broadinstitute.org/

## Supplementary Material

Figure S1 Representation of VCAN isoforms. All isoforms share the same globular domains, G1 (Hyaluronan binding domain) at the amino-terminal, which consists of three domains (Ig like V-type, Link1, Link2), and G3 at the carboxy-terminal, formed by EGF like1, EGF like2 calcium binding, C-type lectin and a complement regulatory protein-like domain (Sushi). The full length protein (isoform V0) contains 2 functional regions glucosaminoglycan attachment domains GAG-α (exon 7) and GAG-β (exon 8). Isoform V1 is similar in structure to isoform V0 but it doesn’t have the GAG-α region, while isoform V2 lacks the GAG-β. V3 is the shortest isoform because it does not contain either GAG-α or GAG-β regions (modified from Figure 2 by Kuwabara et al., 2013).

TABLE S1 Variants statistic according filtering step.

TABLE S2 *In silico* pathogenic prediction of the VCAN H2665L variant. In order to evaluate the pathogenic meaning of the H2665L variant we used different *in silico* tools. This missense variation is not concordantly predicted as pathogenic by *in silico* tools; InterVar defines it as uncertain significance (accordingly to the ACMG/AMP 2015 guideline).

## References

[B1] Bailleul-Forestier I. Molla M. Verloes A. Berdal A. (2008). The genetic basis of inherited anomalies of the teeth. *Part* 1: clinical and molecular aspects of non-syndromic dental disorders. *Eur. J. Med. Genet.* 51 273–291. 10.1016/j.ejmg.2008.02.009 18499550

[B2] Bei M. (2009). Molecular genetics of tooth development. *Curr. Opin. Genet. Dev.* 19 504–510. 10.1016/j.gde.2009.09.002 19875280 PMC2789315

[B3] Burin-des-Roziers C. Rothschild P. R. Layet V. Chen J. M. Ghiotti T. Leroux C. (2017). Deletions overlapping VCAN Exon 8 are new molecular defects for wagner disease. *Hum. Mutat.* 38 43–47. 10.1002/humu.23124 27667122

[B4] Carranza F. A. Newman M. G. (1996). *Clinical Periodontology.* Philadelphia: W.B. Saunders.

[B5] Frazier-Bowers S. A. Vora S. R. (2017). Genetic disorders of dental development: tales from the bony crypt. *Curr. Osteoporos. Rep.* 15 9–17. 10.1007/s11914-017-0342-7 28124261

[B6] Galluccio G. Castellano M. La Monaca C. (2012). Genetic basis of non-syndromic anomalies of human tooth number. *Arch. Oral Biol.* 57 918–930. 10.1016/j.archoralbio.2012.01.005 22325622

[B7] Hou C. Liu Z. X. Tang K. L. Wang M. G. Sun J. Wang J. (2012). Developmental changes and regional localization of Dspp, Mepe, mimecan and versican in postnatal developing mouse teeth. *J. Mol. Histol.* 43 9–16. 10.1007/s10735-011-9368-9 22042093

[B8] Jiang B. Z. Yokohama-Tamaki T. Wang Z. L. Obara N. Shibata S. (2010). Expression, localisation and synthesis of versican by the enamel organ of developing mouse molar tooth germ: an *in vivo* and *in vitro* study. *Arch. Oral Biol.* 55 995–1006. 10.1016/j.archoralbio.2010.07.021 20813348

[B9] Klein O. D. Oberoi S. Huysseune A. Hovorakova M. Peterka M. Peterkova R. (2013). Developmental disorders of the dentition. an update. *Am. J. Med. Genet. C Semin. Med. Genet.* 163C, 318–332. 10.1002/ajmg.c.31382 24124058 PMC3844689

[B10] Kuwabara H. Yoneda M. Isogai Z. (2013). “Expressional alterations of versican, hyaluronan and microfibril associated proteins in the cancer microenvironment,” in *Carcinogenesis*, ed. Tonissen K. (London: IntechOpens).

[B11] Lamperti C. Fang M. Invernizzi F. Liu X. Wang H. Zhang Q. (2012). A novel homozygous mutation in SUCLA2 gene identified by exome sequencing. *Mol. Genet. Metab.* 107 403–408. 10.1016/j.ymgme.2012.08.020 23010432 PMC3490101

[B12] Li R. Yu C. Li Y. Lam T. W. Yiu S. M. Kristiansen K. (2009). SOAP2: an improved ultrafast tool for short read alignment. *Bioinformatics* 25 1966–1967. 10.1093/bioinformatics/btp336 19497933

[B13] Martínez-Moreno J. M. Muñoz-Castañeda J. R. Herencia C. Oca A. M. Estepa J. C. Canalejo R. (2012). In vascular smooth muscle cells paricalcitol prevents phosphate-induced Wnt/β-catenin activation. *Am. J. Physiol. Renal Physiol.* 303 F1136–F1144. 10.1152/ajprenal.00684.2011 22874762

[B14] McKenna A. Hanna M. Banks E. Sivachenko A. Cibulskis K. Kernytsky A. (2010). The genome analysis toolkit: a mapreduce framework for analyzing next-generation DNA sequencing data. *Genome Res.* 20 1297–1303. 10.1101/gr.107524.110 20644199 PMC2928508

[B15] Orsini G. Ruggeri A. Jr. Mazzoni A. Papa V. Mazzotti G. Di Lenarda R. (2007). Immunohistochemical identification of decorin and biglycan in human dentin: a correlative field emission scanning electron microscopy/transmission electron microscopy study. *Calcif. Tissue Int.* 81 39–45. 10.1007/s00223-007-9027-z 17516017

[B16] RuggeriA.OrsiniG.MazzoniA.NatoF.PapaV.PiccirilliM. (2009). Immunohistochemical and biochemical assay of versican in human sound predentine/dentine matrix. *Eur. J. Histochem.* 53 125–133. 10.4081/ejh.2009.e15 19864206

[B17] Schenk R. K. Olah A. J. Herrmann W. (1984). “Preparation of calcified tissues for light microscopy,” in *Methods of Calcified Tissue Preparation*, ed. Dickson G. R. (Amsterdam: Elsevier), 1–56.

[B18] Schindler R. F. Scotton C. Zhang J. Passarelli C. Ortiz-Bonnin B. Simrick S. (2016). POPDC1S201F causes muscular dystrophy and arrhythmia by affecting protein trafficking. *J. Clin. Invest.* 126 239–253. 10.1172/JCI79562 26642364 PMC4701561

[B19] Sone S. Nakamura M. Maruya Y. Takahashi I. Mizoguchi I. Mayanagi H. (2005). Expression of versican and ADAMTS during rat tooth eruption. *J. Mol. Histol.* 36 281–288. 10.1007/s10735-005-5534-2 16200461

[B20] Theocharis A. D. Papageorgakopoulou N. Feretis E. Theocharis D. A. (2002). Occurrence and structural characterization of versican-like proteoglycan in human vitreous. *Biochimie* 84 1237–1243. 10.1016/S0300-9084(02)00015-9 12628301

[B21] Thomas A. S. Branham K. Van Gelder R. N. Daiger S. P. Sullivan L. S. Bowne S. J. (2016). Multimodal imaging in wagner syndrome. *Ophthalmic Surg. Lasers Imaging Retina* 47 574–579. 10.3928/23258160-20160601-10 27327288 PMC5530878

[B22] Zimmermann D. R. Dours-Zimmermann M. T. Schubert M. Bruckner-Tuderman L. (1994). Versican is expressed in the proliferating zone in the epidermis and in association with the elastic network of the dermis. *J. Cell Biol. Mar.* 124 817–825. 10.1083/jcb.124.5.817 8120102 PMC2119961

