# Supplementary figures and images for: Homozygous Recessive Versican Missense Variation Is Associated With Early Teeth Loss in a Pakistani Family
Source: Front Genet. 2019 Jan 21;9:723. doi: 10.3389/fgene.2018.00723 (PMC6357929; doi:10.3389/fgene.2018.00723)

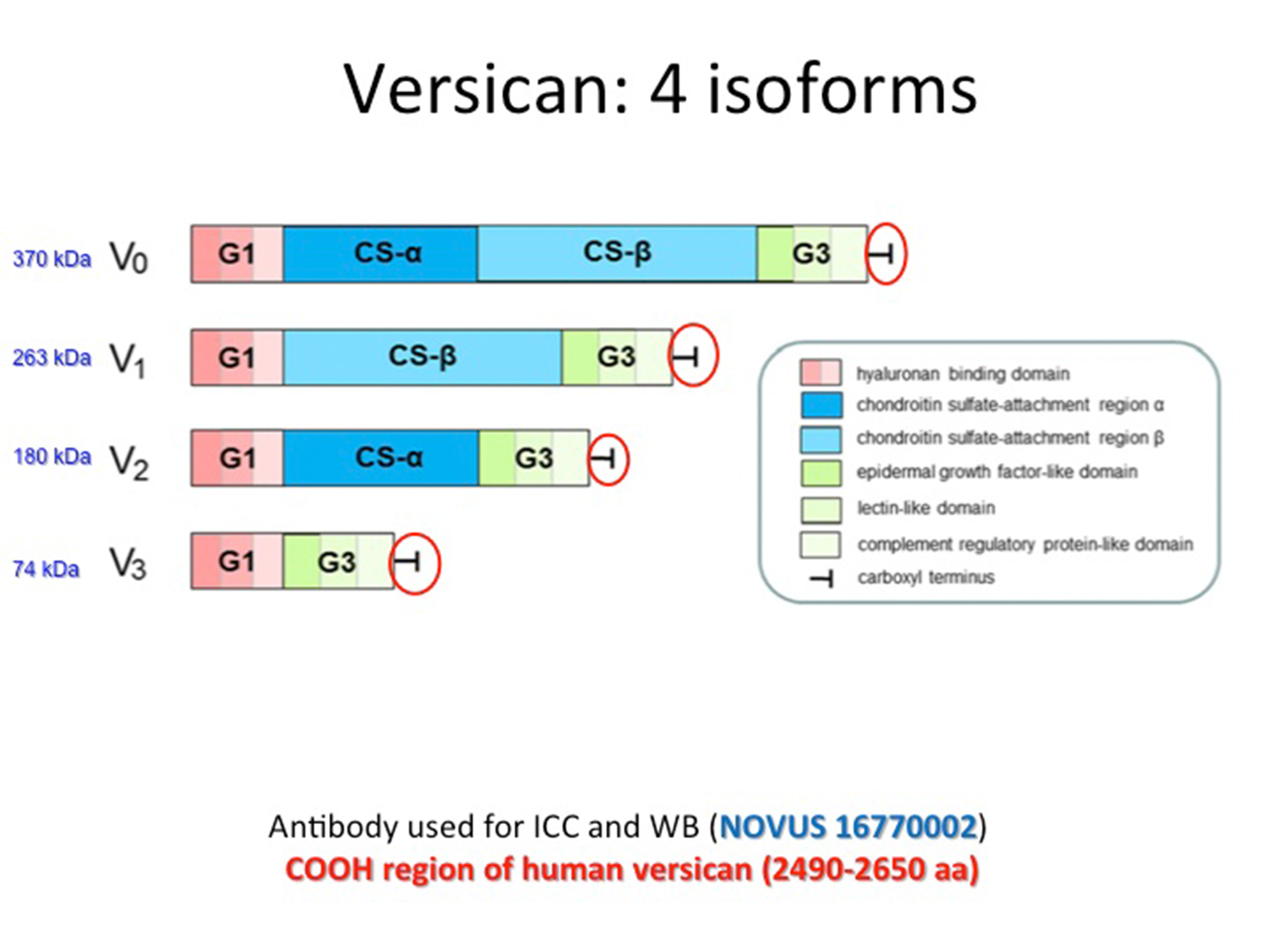

Supplement: Figure S1 — Representation of VCAN isoforms. All isoforms share the same globular domains, G1 (Hyaluronan binding domain) at the amino-terminal, which consists of three domains (Ig like V-type, Link1, Link2), and G3 at the carboxy-terminal, formed by EGF like1, EGF like2 calcium binding, C-type lectin and a complement regulatory protein-like domain (Sushi). The full length protein (isoform V0) contains 2 functional regions glucosaminoglycan attachment domains GAG-α (exon 7) and GAG-β (exon 8). Isoform V1 is similar in structure to isoform V0 but it doesn’t have the GAG-α region, while isoform V2 lacks the GAG-β. V3 is the shortest isoform because it does not contain either GAG-α or GAG-β regions (modified from Figure 2 by Kuwabara et al., 2013). [file Image_1.JPEG]
