# Supplementary material for: Homozygous Recessive Versican Missense Variation Is Associated With Early Teeth Loss in a Pakistani Family
Source: Front Genet. 2019 Jan 21;9:723. doi: 10.3389/fgene.2018.00723 (PMC6357929; doi:10.3389/fgene.2018.00723)
Supplement: TABLE S1 — Variants statistic according filtering step. [file Table_1.docx]

**Supplementary material**

**Supplementary Table 1. Variants statistic according filtering step.**

|  | ***Father_III-1*** | ***Mother_III-2*** | ***Case_IV-1*** | ***Case_IV-3*** |
| --- | --- | --- | --- | --- |
| **Total variants (SNVs & InDels)** | 122262+24123 | 122996+24206 | 119916+23175 | 121893+24064 |
| **MAF<0.5% (1K, ESP, ExAC)** | 27939 | 27451 | 26569 | 27200 |
| **Non-synonymous mutations, alterations at splice site and small insertion/deletions** | 1838 | 1841 | 1852 | 1843 |
| **Excluded variants could be found in In-house database** | 1084 | 1105 | 1097 | 1101 |
| **Shared genes (Homozygous variants for cases, heterozygous for controls)** | 17 Genes | | | |
| **Combined homozygous mapping result (Homozygous region for both affected)** | 14 Genes (*VCAN, RAET1L, SYNE1, TAGAP, C6orf123, MTPAP, RBP3, AGAP6, OR6C74, ITGA7, C15orf48, NARG2, HMG20B, TTC3*) | | | |
| **Causative gene** | ***VCAN*** (c.7994A>T, p.His2665Leu) | | | |
