# Supplementary material for: Homozygous Recessive Versican Missense Variation Is Associated With Early Teeth Loss in a Pakistani Family
Source: Front Genet. 2019 Jan 21;9:723. doi: 10.3389/fgene.2018.00723 (PMC6357929; doi:10.3389/fgene.2018.00723)
Supplement: TABLE S2 — In silico pathogenic prediction of the VCAN H2665L variant. In order to evaluate the pathogenic meaning of the H2665L variant we used different in silico tools. This missense variation is not concordantly predicted as pathogenic by in silico tools; InterVar defines it as uncertain significance (accordingly to the ACMG/AMP 2015 guideline). [file Table_2.docx]

**Supplementary Table 2. In silico pathogenic prediction of the VCAN H2665L variant**

In order to evaluate the pathogenic meaning of the H2665L variant we used different *in silico* tools. This missense variation is not concordantly predicted as pathogenic by *in silico* tools; InterVar defines it as uncertain significance (accordingly to the ACMG/AMP 2015 guideline).

| **IN SILICO TOOLS** | **PREDICTION** | **DELETERIOUS THRESHOLD** |
| --- | --- | --- |
| SIFT | Tolerated (0.12) | Deleterious (<0.05) |
| PolyPhen2_HDIV | Probably damaging (0.974) | Probably damaging (>=0.957), possibly damaging (0.453<=pp2_hdiv<=0.956); benign (<=0.452) |
| PolyPhen2_HVAR | Possibly_damaging (0.459) | Probably damaging (>=0.909),possibly damaging (0.447<=pp2_hdiv<=0.909); benign (<=0.446) |
| MutationTaster | Benign (0.070076) | Deleterious (>0.5) |
| MutationAssessor | Benign (1.79) | Deleterious (>1.938) |
| FATHMM | Deleterious (-1.98) | Deleterious (<-1.5) |
| GERP++ | Benign (2.1) | Deleterious (>4.4) |
| PhyloP | Benign (0.117) | Deleterious (>1.6) |
| SiPhy | Deleterious (12.4059) | Deleterious (>12.17) |
| Gerp | Not conserved (2.1) | Highly conserved (> 5) |
| PhastCons | Benign (0.014) | Deleterious (>0.6) |
| CADD | Benign (10.05) | Deleterious (>15 or MSC >10.73) |
